# Supplementary material for: Hot spot prediction in protein-protein interactions by an ensemble system
Source: BMC Syst Biol. 2018 Dec 31;12(Suppl 9):132. doi: 10.1186/s12918-018-0665-8 (PMC6311905; doi:10.1186/s12918-018-0665-8)
Supplement: Supplementary file 2 — All relevant MATLAB code. A simple Matlab implement of our predictor. (ZIP 19,742 kb) [file 12918_2018_665_MOESM2_ESM.zip › Additional file 2/Read ASA/ASA website/dbMPIKT/4P4QB.html]

 NetSurfP - Protein Surface Accessibility and Secondary Structure Predictions

  

|  |  |  |
| --- | --- | --- |
|  |  | NetSurfP - Protein Surface Accessibility and Secondary Structure Predictions  Technical University of Denmark |

  


---

```
# For publication of results, please cite:
# A generic method for assignment of reliability scores applied to solvent accessibility predictions.
# Bent Petersen, Thomas Nordahl Petersen, Pernille Andersen, Morten Nielsen and Claus Lundegaard
# BMC Structural Biology 2009, 9:51 doi:10.1186/1472-6807-9-51
#
# Column 1: Class assignment - B for buried or E for Exposed - Threshold: 25% exposure, but not based on RSA
# Column 2: Amino acid
# Column 3: Sequence name
# Column 4: Amino acid number
# Column 5: Relative Surface Accessibility - RSA
# Column 6: Absolute Surface Accessibility
# Column 7: Z-fit score for RSA prediction
# Column 8: Probability for Alpha-Helix
# Column 9: Probability for Beta-strand
# Column 10: Probability for Coil
E G  4P4QB                  1    0.724  56.979  -1.520   0.000   0.001   0.999
E S  4P4QB                  2    0.544  63.792   1.017   0.058   0.017   0.925
E A  4P4QB                  3    0.376  41.479  -0.315   0.923   0.002   0.076
E K  4P4QB                  4    0.643 132.306   1.419   0.858   0.002   0.139
E K  4P4QB                  5    0.445  91.475   1.973   0.858   0.002   0.139
B G  4P4QB                  6    0.076   5.973  -0.618   0.938   0.007   0.055
E A  4P4QB                  7    0.369  40.609   1.866   0.970   0.001   0.030
E T  4P4QB                  8    0.573  79.517   2.008   0.970   0.001   0.030
B L  4P4QB                  9    0.124  22.704   1.387   0.970   0.001   0.030
B F  4P4QB                 10    0.063  12.704   0.226   0.970   0.001   0.030
E K  4P4QB                 11    0.503 103.405   1.764   0.923   0.002   0.076
E T  4P4QB                 12    0.548  75.952   1.117   0.782   0.003   0.216
E R  4P4QB                 13    0.417  95.539   1.140   0.502   0.002   0.495
B C  4P4QB                 14    0.061   8.508  -0.058   0.257   0.016   0.727
E L  4P4QB                 15    0.366  66.960   0.680   0.278   0.093   0.628
E Q  4P4QB                 16    0.369  65.850   0.131   0.307   0.165   0.527
B C  4P4QB                 17    0.088  12.341   0.433   0.125   0.227   0.648
B H  4P4QB                 18    0.129  23.556   0.159   0.064   0.216   0.721
E T  4P4QB                 19    0.407  56.382   0.094   0.056   0.142   0.802
B V  4P4QB                 20    0.176  27.082   0.589   0.056   0.142   0.802
E E  4P4QB                 21    0.486  84.904   0.802   0.018   0.088   0.893
E K  4P4QB                 22    0.632 129.982   0.051   0.018   0.047   0.935
E G  4P4QB                 23    0.679  53.477  -1.264   0.018   0.019   0.964
E G  4P4QB                 24    0.416  32.771  -1.174   0.018   0.019   0.964
E P  4P4QB                 25    0.644  91.313   0.023   0.018   0.019   0.964
E H  4P4QB                 26    0.481  87.421  -1.409   0.018   0.047   0.935
E K  4P4QB                 27    0.399  82.136  -0.677   0.019   0.141   0.840
B V  4P4QB                 28    0.224  34.490  -1.271   0.019   0.141   0.840
B G  4P4QB                 29    0.168  13.245  -1.042   0.005   0.045   0.951
B P  4P4QB                 30    0.136  19.369  -0.469   0.018   0.019   0.964
E N  4P4QB                 31    0.300  43.993  -0.332   0.018   0.019   0.964
B L  4P4QB                 32    0.069  12.671  -0.384   0.113   0.043   0.844
E H  4P4QB                 33    0.323  58.808   0.035   0.184   0.043   0.773
B G  4P4QB                 34    0.234  18.408  -1.257   0.278   0.093   0.628
B I  4P4QB                 35    0.069  12.728  -0.026   0.307   0.165   0.527
B F  4P4QB                 36    0.200  40.040  -0.873   0.307   0.165   0.527
E G  4P4QB                 37    0.349  27.458  -1.086   0.113   0.087   0.800
B R  4P4QB                 38    0.203  46.418   0.475   0.113   0.087   0.800
E H  4P4QB                 39    0.502  91.277   0.007   0.191   0.086   0.723
E S  4P4QB                 40    0.334  39.145  -0.491   0.199   0.152   0.649
B G  4P4QB                 41    0.279  21.942  -1.476   0.118   0.150   0.732
E Q  4P4QB                 42    0.358  63.885  -0.890   0.113   0.087   0.800
B A  4P4QB                 43    0.247  27.230  -0.553   0.113   0.087   0.800
E E  4P4QB                 44    0.659 115.057  -1.265   0.053   0.043   0.903
E G  4P4QB                 45    0.562  44.253  -2.105   0.018   0.047   0.935
B Y  4P4QB                 46    0.159  34.085  -0.902   0.052   0.084   0.864
E S  4P4QB                 47    0.479  56.104  -0.340   0.113   0.087   0.800
B Y  4P4QB                 48    0.161  34.363  -0.606   0.184   0.043   0.773
B T  4P4QB                 49    0.190  26.367  -1.379   0.354   0.048   0.598
E D  4P4QB                 50    0.533  76.863  -0.100   0.622   0.015   0.363
B A  4P4QB                 51    0.216  23.814  -1.819   0.622   0.015   0.363
B N  4P4QB                 52    0.107  15.592  -0.804   0.622   0.015   0.363
E I  4P4QB                 53    0.465  86.081  -0.133   0.622   0.015   0.363
E K  4P4QB                 54    0.667 137.243  -0.587   0.522   0.016   0.462
E K  4P4QB                 55    0.389  79.997  -0.504   0.257   0.016   0.727
E N  4P4QB                 56    0.569  83.360  -1.672   0.058   0.017   0.925
B V  4P4QB                 57    0.216  33.184   0.046   0.052   0.084   0.864
E L  4P4QB                 58    0.404  74.046   0.093   0.020   0.205   0.775
B W  4P4QB                 59    0.137  32.973   0.781   0.004   0.138   0.858
E D  4P4QB                 60    0.306  44.109   1.010   0.005   0.045   0.951
E E  4P4QB                 61    0.427  74.579   0.230   0.923   0.002   0.076
E N  4P4QB                 62    0.633  92.613   0.501   0.923   0.002   0.076
B N  4P4QB                 63    0.180  26.367   1.678   0.970   0.001   0.030
B M  4P4QB                 64    0.016   3.262   1.503   0.970   0.001   0.030
E S  4P4QB                 65    0.309  36.262   1.745   0.970   0.001   0.030
E E  4P4QB                 66    0.426  74.352   1.950   0.970   0.001   0.030
B Y  4P4QB                 67    0.056  11.967   1.025   0.923   0.002   0.076
B L  4P4QB                 68    0.051   9.375   0.268   0.858   0.002   0.139
E T  4P4QB                 69    0.486  67.380   1.242   0.694   0.003   0.303
E N  4P4QB                 70    0.333  48.810   0.803   0.176   0.004   0.820
B P  4P4QB                 71    0.086  12.218  -0.392   0.109   0.005   0.886
E K  4P4QB                 72    0.628 129.241   0.816   0.109   0.005   0.886
E K  4P4QB                 73    0.554 113.958   0.341   0.115   0.016   0.868
B Y  4P4QB                 74    0.291  62.101  -0.420   0.113   0.043   0.844
B I  4P4QB                 75    0.269  49.728   0.283   0.052   0.084   0.864
E P  4P4QB                 76    0.527  74.795  -1.038   0.018   0.088   0.893
E G  4P4QB                 77    0.562  44.222  -1.888   0.018   0.047   0.935
B T  4P4QB                 78    0.155  21.429  -2.765   0.018   0.088   0.893
E K  4P4QB                 79    0.368  75.739  -0.375   0.018   0.088   0.893
B M  4P4QB                 80    0.102  20.390  -0.511   0.018   0.047   0.935
E A  4P4QB                 81    0.341  37.600  -0.862   0.018   0.047   0.935
B F  4P4QB                 82    0.265  53.165  -1.705   0.018   0.019   0.964
E G  4P4QB                 83    0.484  38.114  -0.420   0.018   0.019   0.964
E G  4P4QB                 84    0.585  46.071  -1.241   0.005   0.015   0.979
B L  4P4QB                 85    0.103  18.841  -0.141   0.005   0.015   0.979
E K  4P4QB                 86    0.444  91.393  -0.295   0.005   0.015   0.979
E K  4P4QB                 87    0.561 115.459   1.430   0.003   0.003   0.994
E E  4P4QB                 88    0.572  99.946  -0.047   0.858   0.002   0.139
E K  4P4QB                 89    0.623 128.110   1.032   0.923   0.002   0.076
B D  4P4QB                 90    0.188  27.091   0.719   0.923   0.002   0.076
B R  4P4QB                 91    0.073  16.763   1.082   0.970   0.001   0.030
E N  4P4QB                 92    0.371  54.300   0.767   0.970   0.001   0.030
B D  4P4QB                 93    0.232  33.402   1.626   0.970   0.001   0.030
B L  4P4QB                 94    0.019   3.534   1.591   0.970   0.001   0.030
B I  4P4QB                 95    0.084  15.614   1.275   0.988   0.000   0.012
E T  4P4QB                 96    0.279  38.725   1.264   0.988   0.000   0.012
B Y  4P4QB                 97    0.140  29.918   1.312   0.970   0.001   0.030
B L  4P4QB                 98    0.056  10.180   0.821   0.970   0.001   0.030
E K  4P4QB                 99    0.380  78.125   1.575   0.923   0.002   0.076
E K  4P4QB                100    0.472  97.029   1.063   0.858   0.002   0.139
B A  4P4QB                101    0.196  21.610   0.467   0.600   0.003   0.397
E C  4P4QB                102    0.458  64.317  -0.389   0.246   0.004   0.750
E E  4P4QB                103    0.888 155.151   1.283   0.003   0.003   0.994

Explain the output.  Go back.
```

---
